# Glycemic and Insulinemic Responses of Healthy Humans to a Nutrition Bar with or without Added Fibersym^®^ RW, a Cross-Linked Phosphorylated RS4-Type Resistant Wheat Starch

**DOI:** 10.3390/ijerph192113804

**Published:** 2022-10-24

**Authors:** Trevor J. Steele, Catherine C. Steele, Clodualdo C. Maningat, Paul A. Seib, Mark D. Haub, Sara K. Rosenkranz

**Affiliations:** 1 Department of Food, Nutrition, Dietetics and Health, Kansas State University, Manhattan, KS 66506, USA; 2 Physical Activity and Nutrition Clinical Research Consortium, Kansas State University, Manhattan, KS 66506, USA; 3 MGP Ingredients Inc., Atchison, KS 66002, USA; 4 Grain Science and Industry Emeritus, Kansas State University, Manhattan, KS 66506, USA; 5 Department of Kinesiology and Nutrition Sciences, University of Nevada, Las Vegas, NV 89154, USA

**Keywords:** resistant starch type-4, RS4, glycemic response, insulinemic response

## Abstract

The current study compared postprandial glycemic and insulinemic responses to four nutrition bars containing two different doses of resistant starch type-4. Normoglycemic adults (*n* = 17) completed six treatments, consuming either 50 g or 30 g digestible carbohydrate as: dextrose beverages (DEX), control puffed wheat bars (PWB), or RS4 test bars (RS4). Glucose (mg/dL) and insulin (µIU/mL) were measured at baseline and 10, 20, 30, 60, 90, and 120 min. There was a main effect of dose and treatment on glucose incremental area under the curve (iAUC, *ps* < 0.001), such that RS4 (50 g: 941, 95% confidence interval (CI): 501, 1519; 30 g: 481, 95% CI: 186, 914) was lower than PWB (50 g: 1746, 95% CI: 1109, 2528; 30 g: 693, 95% CI: 331, 1188) and DEX (50 g: 1940, 95% CI: 1249, 2783; 30 g:1432, 95% CI: 883, 2114). There was a main effect of dose and treatment on insulin iAUC (*ps* < 0.001), such that RS4 (50 g: 1993, 95% CI: 1347, 2764; 30 g: 943, 95% CI: 519, 1493) was lower than PWB (50 g: 3501, 95% CI: 2625, 4502; 30 g: 1789, 95% CI: 1193, 256) and DEX (50 g: 3143, 95% CI: 2317, 4095; 30 g: 2184, 95% CI: 1519, 2970). Results demonstrate significantly lower glycemic and insulinemic responses following consumption of nutrition bars containing RS4, regardless of dose, when compared with puffed wheat bars and dextrose.

## 1. Introduction

High dietary fiber consumption has been associated with reduced risk of developing non-communicable chronic disease, including cardiovascular disease and diabetes, among others [1,2]. Further, recent research suggests that dietary fiber may play a role in treating obesity-related disorders [3]. Despite numerous studies showing benefits of dietary fiber, national levels of consumption remain drastically low with 90% of American women and 97% of American men not meeting recommended fiber intake [4]. A recent review suggests that consuming enough dietary fiber, at “advisable” amounts between 30–40 g, has several consistent benefits for human health including “curative and preventive effects for diseases or conditions such as obesity, certain types of cancers, cardiovascular diseases, diabetes, and constipation,” and that including fiber in manufactured carbohydrate containing products has the potential to enhance public health and aid in the prevention of non-communicable chronic disease [5].

One method of adding fiber to food products is through the addition of resistant starch type 4 (RS4), a human-created fiber primarily used as a substitute for flour in food products. Type 4 RS is one of the five classes of starch-based ingredients recognized to resist human digestion [6]. Several forms of RS4 are known where the chemical structure of the starch base has been modified, including esterified or etherified starch, tightly cross-linked starch, pyrodextrins, and recrystallized (re-associated) dextrins made from debranched or acid-hydrolyzed starch. The experimental RS4 tested in this work is a commercial source of cross-linked phosphorylated wheat starch, which has been approved for food labeling as dietary fiber by the US Food and Drug Administration (FDA) [7].

Existing randomized controlled trials have shown beneficial effects of RS4 intake on glycemic and insulinemic responses [8,9,10,11,12,13,14,15,16,17,18]. To test the benefits of RS4 for fiber food label classification, researchers often test at a 50 g amount of digestible carbohydrate [8,13,19]. In a research setting, this amount of digestible carbohydrate typically requires a larger bolus of food than is typically consumed by consumers in a single sitting. For this reason, there may be differential responses observed at these testing amounts that may not translate to a typical, standard serving size for consumers [13]. There is limited research, using an FDA-approved testing approach for fiber food label classification, incorporating lower doses (<50 g) of digestible carbohydrate that are likely to represent more typical consumption amounts. However, a recent randomized controlled trial reported improvements in glycemic and insulinemic responses at a lower amount of digestible carbohydrate (35 g), a dose more typical of a consumer portion, regardless of the dose of RS4 [10].

In order to elucidate the effects of RS4 on postprandial glycemic and insulinemic responses, we conducted an investigation of a cross-linked phosphorylated RS4 resistant wheat starch (Fibersym^®^ RW) provided in the form of a nutrition bar, used in similar studies [8,10], at a high dose (50 g) and a low dose (30 g) of digestible carbohydrate. The primary purpose of this randomized, single-blinded, crossover study was to determine whether glycemic and insulinemic responses were different when using an amount of digestible carbohydrate more commonly used in research as compared with a consumer-friendly portion of digestible carbohydrate more typical of a marketplace food product. In addition, we sought to investigate the glycemic and insulinemic responses to RS4 as compared to a standard puffed wheat bar (PWB) and a dextrose control at both the low and high doses (50 g and 30 g) of digestible carbohydrate. We hypothesized that the Fibersym^®^ RS4 bar would elicit lower glucose and insulin responses compared to the PWB and dextrose control at both matched doses.

## 2. Materials and Methods

### 2.1. Recruitment of Participants

Fifteen apparently healthy participants (ages 20–38 years) with no history of diagnosed health conditions completed this study. A power analysis was conducted using data from Al-Tamimi et al. [8], indicating that a total sample of 12 participants would be needed to detect large effects (d = 1.7) at 80% power and alpha at 0.05 when comparing 50 g digestible carbohydrate PWB and RS4 nutrition bars. In order to account for potential participant dropout, we recruited 17 participants. All participants completed a medical history questionnaire and a screening visit to determine eligibility to participate in this study. Participants were excluded if they had a baseline fasting blood glucose ≥ 100 mg/dL, consumed a diet high in dietary fiber (>50 g/day), were current smokers or had smoked within the past six months, were pregnant or lactating, had a wheat or gluten allergy, or had any diagnosed health conditions that might have affected glycemia or insulinemia. This study was approved by the Institutional Review Board for Research Involving Human Subjects at Kansas State University (IRB #9368) and conformed to the Declaration of Helsinki.

The current study was conducted as a single-blinded randomized-controlled crossover trial, where all participants experienced all treatments. Methods and procedures were modified from Steele et al., [13]. Specifically, the timepoints of whole blood collection and the method of glucose analysis were modified.

All testing sessions were performed in the Physical Activity and Nutrition Clinical Research Consortium (PANCRC) at Kansas State University, Manhattan, Kansas. Prior to randomization, participants were required to pass an initial 2-h screening visit following consumption of a 75 g glucose tolerance dextrose beverage. In order to take part in the study, participants had to have a glucose value < 100 mg/dL at baseline and <140 mg/dL at two hours following consumption of the dextrose beverage. Upon completion of screening, participants were randomly assigned to a series of six treatments via a blocked Latin-square design [20,21]. Participants completed a postprandial assessment following a glucose tolerance test protocol at each visit. The postprandial assessment was conducted as previously described in Steele, Maningat et al. [13]. Each participant consumed one randomized treatment during a given testing session. The six treatments were completed following a 10–12 h fast with at least 48 h between treatments and included the following: (1) 50 g dextrose beverage (50DEX; Trutol^®^50 glucose-tolerance beverage), (2) 30 g dextrose beverage (30DEX), (3) 50 g of digestible carbohydrate from a PWB bar (50PWB), (4) 30 g of digestible carbohydrate from a PWB bar (30PWB), (5) 50 g of digestible carbohydrate from an RS4 bar (50RS4), and (6) 30 g of digestible carbohydrate from an RS4 bar (30RS4). The primary purpose of this study was to determine differences between the two nutritional bars whereas the dextrose beverage was included as a reference for the participants’ insulinemic and glycemic response to carbohydrates without the interaction of other macronutrients. Additional details for the nutrition bar treatments are shown in Table 1. To reduce variability in daily postprandial responses, participants were asked to maintain dietary and physical activity habits throughout the study period. Additionally, participants were asked to record what they ate the night prior to their first treatment and were reminded to consume the same snack/meal prior to subsequent testing sessions. Satiety was measured via the Holt Satiety Questionnaire at baseline, 30, 60, 90, and 120 min during each testing sessions to test levels of satiety throughout the 2 h testing period. Anthropometric data including height (Invicta Plastics), weight (Pelstar LLC), waist circumference using a standard tape measurer, BMI (GE Prodigy, Lunar General Electric), and satiety (Holt Satiety Scale) were conducted as previously described [13]. A Dual-energy X-ray Absorptiometry (DXA) scan was conducted at the final testing session to determine body composition and as a form of compensation for participating.

### 2.2. Blood Analysis

To determine whole blood glucose and plasma insulin, whole blood was collected at baseline, 10, 20, 30, 60, 90, and 120 min following consumption of the randomized treatment. Venous whole blood samples were drawn into a 5 mL syringe (BD), and a small amount was used to measure whole blood glucose using a Cholestech Cassette (Alere Cholestech LDX TC•GLU cassettes, product code 10-988; Waltham, MA, USA). Samples were measured in duplicate, and the mean of two measurements was used for statistical analysis. Blood processing following whole blood glucose analysis was conducted as previously described [13].

The glucose tolerance test beverage provided either 50 g or 30 g of dextrose (Thermo Fischer Scientific, Catalog Number: 401074P; Waltham, MA, USA). The PWB and RS4 bars were provided by MGP Ingredients Inc. (Atchison, KS, USA) and were formulated using the same general ingredients used in a previous study [8]. Thus, all ingredients in the PWB and RS4 bars were identical except for the source of starch used. A breakdown of the nutrient composition per 100 g of nutrition bars is displayed in Table 1. The 50PWB treatment was tested using a 91.7 g puffed wheat bar providing 50 g of digestible carbohydrate from puffed wheat yielding 1126.9 KJ (269.2 kcals). The 50RS4 treatment was tested using a 106.4 g RS4 bar providing 50 g of digestible carbohydrate from RS4 yielding 1017.2 KJ (243.0 kcals). The 30PWB treatment was tested using a 55.0 g puffed wheat bar yielding 676.0 KJ (161.5 kcals) and the 30RS4 treatment was tested using 63.8 g of RS4 bar yielding 609.9 KJ (145.7 kcals), both providing 30 g of digestible carbohydrate. All ingredients were food grade and generally recognized as safe. The PWB and RS4 bars were distinguishable from one another as the puffed wheat had a puffed appearance compared to the non-puffed nature of RS4. Participants were not aware of the specifics of the treatment being consumed (i.e., which contained RS4, and which did not) until all nutrition bar treatments had been completed.

### 2.3. Statistical Analysis

Data analyses were performed using R, version 3.5.3 [22]. A repeated-measures linear regression model was used to determine differences between dose amounts (Dose), treatment type (Treatment), and the interaction between dose and all treatments. Treatment, Dose, and Treatment × Dose were included as fixed effects. Treatment (DEX, PWB, RS4) and Dose (50 g digestible carbohydrate, 30 g digestible carbohydrate) were effect coded. Participant was included as a random effect to account for individual differences between treatment. Statistical significance was set at *p* < 0.05. Posthoc comparisons were performed for significant effects with the emmeans package in R [23]. The Results section outlines the results from the posthoc comparisons (results of the full analysis are reported in the  supplementary file). Outcome variables were transformed for normality according to boxcox analysis using the MASS package in R [24]. Following the transformation of data, histograms and residual plots were used to verify the normal distribution of data. If more than one transformation was identified using the boxcox analysis [24], the transformation used was aligned across similar variables. The analytic parameters and calculations for peak values and homeostatic model assessment of insulin resistance (HOMA-IR) are described in Steele et al. [13]. Glucose and insulin incremental area under the curve (iAUC) were calculated in R using the trapezoid method [25]. Glucose iAUC and insulin iAUC were square-root transformed. Glucose peak, insulin peak, glucose time-to-peak, insulin time-to-peak, glucose baseline-to-peak, insulin baseline-to-peak, insulin:glucose ratio, and HOMA-IR were log-transformed. Estimated values were converted back to the original units using the emmeans package in R and are reported in the text and tables and raw data are graphed. Concealment of the treatments was not performed due to visible differences in treatment conditions. To reduce bias, analysis was performed by a different researcher than the person administering the visits. Sequence of treatments received was not included in the statistical analysis because participants were randomly assigned to treatments using a blocked Latin square design, which is intended to control for the effect of the sequence [20,21]. Further, the study was not adequately powered or intended to investigate the effect of sequence.

## 3. Results

**Figure 1:**
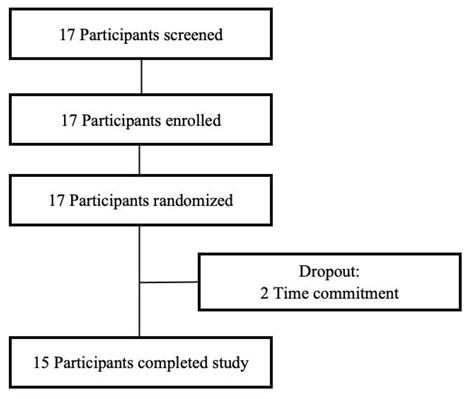
Consort Diagram.

Fifteen apparently healthy adults (8 male; 7 female) between the ages of 20–38 years, with no diagnosed health conditions, completed this study. A total of two participants withdrew from the study following one and two sessions (Figure 1). Demographic, anthropometric, baseline glucose, and baseline insulin values are shown in Table 2. Seven subjects were classified as overweight or obese (BMI ≥ 25.0 kg/m^2^) and the remainder were classified as normal weight (<25.0 kg/m^2^).

**Figure 2:**
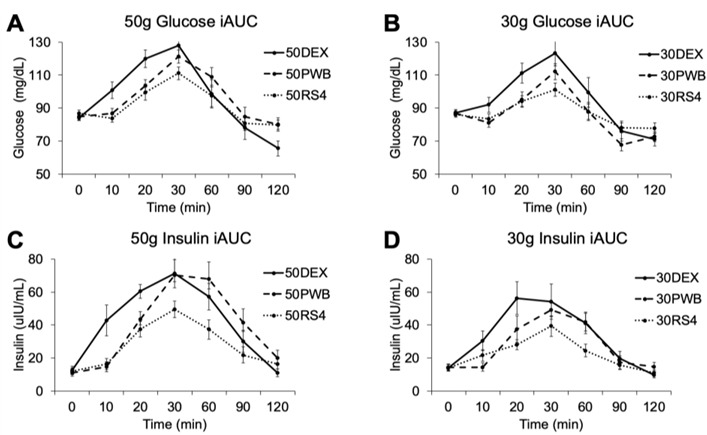
Postprandial glycemic and insulinemic responses to dextrose PWB and RS4 bars during the 2 hr testing period. Data represent means and standard errors. (**A**,**B**) report on glucose iAUC for the 50 g and 30 g treatments, respectively. (**C**,**D**) report on insulin iAUC for the 50 g and 30 g treatments, respectively.

Means and 95% confidence intervals for dose and treatment comparisons are reported in Table 3. Postprandial glucose and insulin data for each treatment are displayed in Figure 2. There was a significant main effect of dose and treatment for glucose iAUC, insulin iAUC, glucose peak, insulin peak, glucose baseline-to-peak, and insulin baseline-to-peak. Specifically, 30 g responses were significantly lower than 50 g responses.

In addition, RS4 treatment responses were significantly lower compared with PWB and DEX responses at both the 30 g and 50 g doses. There was no significant interaction for any of the outcome measures; the means and CIs of each treatment for each dose are reported in Table 4.

For HOMA-IR, there was a significant main effect of dose, *p* < 0.001, such that the 50 g dose resulted in lower HOMA-IR values compared with 30 g doses. There was no effect of treatment, dose, or an interaction for insulin to glucose ratio (*ps* > 0.05). No differences were observed when comparing satiety across the three groups (*ps* > 0.05).

## 4. Discussion

Given the prevalence of non-communicable chronic diseases that may be partially mitigated by increasing fiber consumption and the promising preliminary research on resistant starch type 4 [8,10,11,12,13,14,15,16,17,18], the primary aim of this study was to investigate the effects of two doses of digestible carbohydrate combined with Fibersym^®^ RW (RS4) on glycemic and insulinemic responses when compared with puffed wheat bar (PWB) and dextrose. The current study utilized the FDA-approved testing method of matching digestible carbohydrates to determine whether glycemic and insulinemic responses were similar using a 50 g dose of digestible carbohydrate in addition to a 30 g dose of digestible carbohydrate, representing an amount similar to a typical serving size a consumer may experience daily [26,27]. Significantly lower glucose and insulin iAUC were observed at both doses of RS4 when compared to a puffed wheat bar and a dextrose beverage. These results suggest that potential beneficial metabolic responses for nutrition bars containing RS4 are consistent in larger as well as lower doses of digestible carbohydrate that are more in line with the carbohydrate content for serving sizes of nutrition bar products purchased by consumers.

Secondary outcomes followed a similar pattern, indicating lower values following RS4 consumption for glucose and insulin peak as well as glucose and insulin baseline-to-peak when compared with PWB and Dextrose. These reduced postprandial values, considered alongside the reductions in glucose and insulin iAUC, indicate consistent glycemic and insulinemic reductions following consumption of RS4 nutrition bars as compared with PWB and dextrose control beverages matched for digestible carbohydrate.

Research investigating multiple RS4 doses within a single study is scarce. Of the research that is available, inconsistent results are reported for glycemic and insulinemic responses when matching for digestible carbohydrate [10,19]. A recent study suggests that there are no differences in glycemic or insulinemic responses across different doses of RS4 when matching for digestible carbohydrate [10,19], consistent with Steele et al. [13]. However, the results here demonstrate beneficial effects of RS4 consumption on postprandial glycemic and insulinemic responses regardless of the amount of digestible carbohydrate consumed in an amount between 30–50 g.

Du et al., used the addition method, where the RS4 dose was added on top of the formulated control product; they did not find a significantly lower glycemic or insulinemic response for the RS4 cereal bar [19]. However, Gourineni et al., using the substitution method, where the RS4 replaced (was substituted for) a similar ingredient in the control product and the tested nutrition bars, matched control and test nutrition bars for digestible carbohydrate, similar to the current study, and found a reduction in postprandial blood glucose and insulin by RS4 [10]. This reduction in postprandial glycemic and insulinemic response agrees with the results observed in Al-Tamimi et al. [8]. The studies that found no differences between RS4 and the control treatments used the FDA approved add-on method, while the studies that found differences between RS4 and the control treatments used the FDA approved substitution method [27].

Results from the current study, along with the available results from previous investigations of differential responses following multiple RS4 doses, elucidates two potentially important factors to note regarding the impact of RS4 on glycemic and insulinemic responses. The addition of RS4 on top of a given carbohydrate-containing product does not appear to negate the glycemic and insulinemic responses to other existing carbohydrate ingredients in the formulated nutrition bar. However, when the non-fiber ingredient is substituted with RS4, reductions in glycemic and insulinemic responses appear. Thus, the method of inclusion of RS4 in a given product, addition to a product vs. substituted in a product, may be important for attenuations in glycemic and insulinemic responses to exist. Therefore, the preparation of the food product may be important when considering the potential benefits of an RS4 product as compared to a standard non-RS4 carbohydrate containing product.

The current study has several strengths, including the use of a randomized single-blind crossover design. This study design allowed for results to be parsed apart for both doses of digestible carbohydrate among all three treatment types, all while reducing between subject heterogeneity with the cross over design. An additional strength of this study is the use of a single method of product formulation for all nutrition bar treatments. For the current study, we used the substitution method, matching for digestible carbohydrate across dose and treatment type. This method eliminates many potential limitations of the interpretation of results when comparing doses, treatment types, and Dose x Treatment interactions due to the absence of confounding interactions occurring between multiple carbohydrate sources observed using the add-on method. One limitation of the current study is the use of multiple comparisons, which inherently increases the potential occurrence of a type 1 error. A second limitation was the finding of a significant effect of dose for HOMA-IR, a measure based on fasting values that should not be influenced by the experimental treatments, indicating a type 1 error. We designed the study to account for daily differences in baseline values via the randomized crossover design, an appropriately powered sample size, a consistent 2–7 day washout period, and by adding a control for the last consumed food the night prior to each visit. Our hypothesis was that participants may not have fully abided by the controlled meal the night prior to each visit, and/or may have engaged in different levels of physical activity, thereby affecting energy balance, which may play a role in baseline differences in HOMA-IR between treatments [28]. Alternatively, weight was not strictly monitored and may have affected the HOMA-IR results. However, body composition was not expected to change given the short-term nature of the study and the requirement to maintain exercise and diet. A third limitation was the single blinded nature of the study along with the lack of treatment concealment during data analysis.

## 5. Conclusions

Results from the current study indicate similar and consistent reductions in glycemic and insulinemic responses across doses typically used in FDA fiber food label testing (50 g) as well as doses more typically experienced by consumers (30 g). These findings suggest that substitution of RS4 in carbohydrate containing products may be a potentially feasible approach for helping to mitigate the increasing burden of non-communicable chronic diseases. In order to evaluate the previous conflicting RS4 evidence regarding glycemic and insulinemic responses to RS4 consumption, future research should investigate differences between FDA approved and non-FDA approved testing methods, and multiple doses of RS4 consumption utilizing FDA approved testing methods. In addition, further research should investigate long-term, well powered human intervention studies to understand the role of RS4 in modulating human health.

## Figures and Tables

**Table 1:** Nutrient composition of PWB and RS4 bar treatments.

Component	50PWB	50RS4	30PWB	30RS4
Ash (g)	1.7	1.7	1.1	0.9
Moisture (g)	14.9	16.9	8.9	10.0
Carbohydrate (g)	62.0	79.7	37.3	47.8
Digestible carbohydrate (g) ^1^	50.0	50.0	30.0	30.0
Dietary fiber (g)	12.0	29.7	7.3	17.9
Fat (g)	3.3	2.1	2.0	1.3
Protein (g)	9.9	6.0	5.8	3.6
Total calories (KJ) (kcal)	1126.9 (269.2)	1017.2 (243.0)	676.0 (161.5)	609.9 (145.7)
Weight provided to participants (g)	91.7	106.4	55.0	63.8

^1^ Digestible carbohydrate was calculated as Carbohydrate (g) minus dietary fiber (g).

**Table 2:** Baseline participant characteristics of individuals who completed the study. Results are reported as Mean ± SD.

	All (*n* = 15)
Sex (Male;Female)	8;7
Age (years)	26.1 ± 4.8
Height (cm)	174.0 ± 8.8
Weight (kg)	76.1 ± 16.8
BMI (kg/m^2^)	24.9 ± 4.0
Body Fat (DXA, %) ^1^	23.6 ± 9.3
Fasting Glucose (mg/dL)	84.0 ± 5.77
Fasting Insulin (μIU/mL)	12.6 ± 9.29

^1^ DXA: Dual-energy X-ray Absorptiometry scan for body fat percentage.

**Table 3:** Summary of means for treatments and doses. Values are reported as mean estimates and 95% confidence interval. Means reported are obtained from the posthoc comparisons of the repeated measures linear regression.

		Treatment			Dose		
	Variable	DEX	PWB	RS4	30 g	50 g	*p*
Glucose	iAUC (mg/dL × 2 h)	1677 * (1134, 2325)	1160 * (721, 1703)	692 (365, 1123)	824 (480, 1260)	1508 (1020, 2093)	<0.001
	Peak (mg/dL)	132 * (124, 142)	119 * (111, 127)	108 (101, 115)	115 (108, 122)	123 (116, 132)	0.01
	Baseline-to-peak (mg/dL)	41.1 * (29.2, 58.0)	27.8 * (19.8, 39.1)	17.1 (12.2, 24.1)	22.0 (16.1, 30.0)	33.1 (24.0, 45.5)	0.005
	Time-to-peak (Minutes)	27.5 (23.3, 32.6)	34.3 (29.0, 40.5)	32.0 (27.0, 37.8)	32.9 (28.3, 38.3)	29.5 (25.2, 34.4)	0.12
Insulin	iAUC (μIU/mL × 2 h)	2642 * (1993, 3382)	2574 * (1934, 3305)	1419 (953, 1978)	1593 (1126, 2142)	2840 (2198, 3564)	<0.001
	Peak (μIU/mL)	67.3 * (54.8, 82.7)	62.2 * (50.6, 76.4)	45.6 (37.1, 56.0)	51.0 (41.9, 62.1)	65 (53.3, 79.2)	<0.001
	Baseline-to-peak (μIU/mL)	54.4 * (43.8, 67.4)	50.6 * (40.9, 62.5)	31.3 (25.3, 38.8)	35.8 (29.5, 43.4)	54.5 (44.6, 66.4)	<0.001
	Time-to-peak (Minutes)	27.6 (23.3, 32.7)	38.6 (32.7, 45.6)	33.0 (27.9, 39.0)	31.3 (27.2, 35.9)	34.3 (29.6, 39.8)	0.30
Indexes	HOMA-IR ^1^	2.50 (1.88, 3.31)	2.34 (1.77, 3.10)	2.40 (1.81, 3.18)	2.66 (2.03, 3.49)	2.18 (1.66, 2.87)	0.01
	Insulin:Glucose ^2^	1.95 (1.09, 3.49)	3.01 (1.69, 5.36)	2.75 (1.54, 4.89)	2.55 (1.48, 4.39)	2.50 (1.44, 4.34)	0.92

For treatment, comparisons with RS4 are reported where * = *p* < 0.05. ^1^ HOMA-IR was used to determine insulin resistance. ^2^ Insulin-to-glucose ratio was used to determine impaired glucose tolerance.

**Table 4:** Summary of overall means for each treatment at each dose. Values are reported as mean estimates and 95% confidence intervals. Means reported are obtained from the posthoc comparisons of the repeated measures linear regression.

		30 g			50 g		
	Variable	DEX	PWB	RS4	DEX	PWB	RS4
Glucose	iAUC (mg/dL × 2 h)	1432 * (883, 2114)	693 * (331, 1188)	481 (186, 914)	1940 * (1249, 2783)	1746 * (1109, 2528)	941 (501, 1519)
	Peak (mg/dL)	130 * (120, 140)	114 * (105, 123)	103 (95.5, 112)	135 * (124,147)	125 * (114.7, 135)	112 (103.2, 121)
	Baseline-to-peak (mg/dL)	34.5 * (23.1, 51.5)	22.0 * (14.7, 32.8)	14.0 (9.3, 21.1)	49.0 * (31.7, 75.8)	35.1 * (23.0, 53.7)	21.0 (13.9, 31.6)
	Time-to-peak (Minutes)	31.8 * (26.1, 38.7)	32.6 * (26.8, 39.8)	34.3 (28.0, 42.1)	23.9 * (19.2, 29.7)	36 * (29.2, 44.4)	29.7 (24.2, 36.4)
Insulin	iAUC (μIU/mL × 2 h)	2184 * (1519, 2970)	1789 * (1193, 2506)	943 (519, 1493)	3143 * (2317, 4095)	3501 * (2625, 4502)	1993 (1347, 2764)
	Peak (μIU/mL)	59.1 * (47.0, 74.3)	55.4 * (44.0, 69.6)	40.6 (32.1, 51.2)	76.8 * (60.8, 96.9)	69.8 * (55.3, 88.2)	51.2 (40.5, 64.6)
	Baseline-to-peak (μIU/mL)	45.7 * (35.4, 58.8)	40.7 * (31.6, 52.4)	24.7 (19.1, 32.1)	64.7 * (49.1, 85.4)	62.9 * (48.1, 82.2)	39.7 (30.4, 52.9)
	Time-to-peak (Minutes)	29.5 * (23.8, 36.7)	36.1 * (29.1, 44.8)	28.7 (22.9, 35.9)	25.8 * (20.3, 32.9)	41.3 * (32.7, 52.0)	38 (30.1, 47.9)
Indexes	HOMA-IR ^1^	2.64 (1.94, 3.5)	2.70 (1.98, 3.67)	2.65 (1.95, 3.60)	2.36 (1.73, 3.23)	2.03 (1.50, 2.77)	2.17 (1.59, 2.96)
	Insulin:Glucose ^2^	1.98 (1.03, 3.80)	3.48 (1.81, 6.68)	2.41 (1.24, 4.69)	1.92 (0.95, 3.86)	2.60 (1.32, 5.15)	3.13 (1.61, 6.10)

Within each dose, comparisons with RS4 are reported where * = *p* < 0.05. ^1^ HOMA-IR was used to determine insulin resistance. ^2^ Insulin-to-glucose ratio was used to determine impaired glucose tolerance.

## Data Availability

The data that support the findings of this study are available on request from the corresponding author T.J.S. The data are not publicly available due to third party restrictions. The data, however, are available from the authors upon reasonable request and with permission of MGP Ingredients.

## Supplementary Materials

The following supporting information can be downloaded at: https://www.mdpi.com/article/10.3390/ijerph192113804/s1.
