# Supplementary material for: Glycemic and Insulinemic Responses of Healthy Humans to a Nutrition Bar with or without Added Fibersym® RW, a Cross-Linked Phosphorylated RS4-Type Resistant Wheat Starch
Source: Int J Environ Res Public Health. 2022 Oct 24;19(21):13804. doi: 10.3390/ijerph192113804 (PMC9654973; doi:10.3390/ijerph192113804)
Supplement: Supplementary file 1 [file ijerph-19-13804-s001.zip › ijerph-1948985-supplementary.pdf]

```
GaucModel<-lmer(sqrt(GlucoseiAUC)~Dose*Treatment + (1|Subject),data=ba
rData)
anova(GaucModel)
```

```
## Type III Analysis of Variance Table with Satterthwaite's method
##                 Sum Sq Mean Sq NumDF  DenDF F value    Pr(>F)
## Dose           2079.83 2079.83     1 63.693 16.0699 0.0001626 ***
## Treatment      2880.66 1440.33     2 63.558 11.1288  7.18e-05 ***
## Dose:Treatment  310.52  155.26     2 63.517  1.1996 0.3080386
## ---
## Signif. codes:  0 '***' 0.001 '**' 0.01 '*' 0.05 '.' 0.1 ' ' 1
```

```
IaucModel<-lmer(sqrt(InsuliniAUC)~Dose*Treatment + (1|Subject),data=ba
rData)
anova(IaucModel)
```

```
## Type III Analysis of Variance Table with Satterthwaite's method
##                Sum Sq Mean Sq NumDF  DenDF F value    Pr(>F)
## Dose           3812.7  3812.7     1 66.123 30.2329 6.644e-07 ***
## Treatment      3359.7  1679.8     2 66.139 13.3202 1.377e-05 ***
## Dose:Treatment  207.9   104.0     2 66.139  0.8243     0.443
## ---
## Signif. codes:  0 '***' 0.001 '**' 0.01 '*' 0.05 '.' 0.1 ' ' 1
```

```
GPeakModel<-lmer(log(GlucosePeak)~Dose*Treatment + (1|Subject),data=ba
rData)
anova(GPeakModel)
```

```
## Type III Analysis of Variance Table with Satterthwaite's method
##                 Sum Sq  Mean Sq NumDF  DenDF F value    Pr(>F)
## Dose           0.09933 0.099326     1 63.739  6.9529    0.0105 *
## Treatment      0.57865 0.289325     2 63.563 20.2530 1.567e-07 ***
## Dose:Treatment 0.00994 0.004969     2 63.509  0.3479    0.7075
## ---
## Signif. codes:  0 '***' 0.001 '**' 0.01 '*' 0.05 '.' 0.1 ' ' 1
```

```
IPeakModel<-lmer(log(InsulinPeak)~Dose*Treatment + (1|Subject),data=ba
rData)
anova(IPeakModel)
```

```
## Type III Analysis of Variance Table with Satterthwaite's method
##                 Sum Sq Mean Sq NumDF  DenDF F value    Pr(>F)
## Dose           1.25096 1.25096     1 66.076 13.7214 0.0004351 ***
```

```
## Treatment      2.39074 1.19537     2 66.088 13.1117   1.6e-05 ***
## Dose:Treatment 0.00427 0.00214     2 66.088  0.0234 0.9768561
## ---
## Signif. codes:  0 '***' 0.001 '**' 0.01 '*' 0.05 '.' 0.1 ' ' 1
```

```
GTTPModel<-lmer(log(GlucoseTtP)~Dose*Treatment + (1|Subject),data=barD
ata)
anova(GTTPModel)
```

```
## Type III Analysis of Variance Table with Satterthwaite's method
##                 Sum Sq Mean Sq NumDF  DenDF F value  Pr(>F)
## Dose           0.24875 0.24875     1 64.204  2.4799 0.12022
## Treatment      0.67032 0.33516     2 64.004  3.3413 0.04166 *
## Dose:Treatment 0.51079 0.25539     2 63.943  2.5461 0.08631 .
## ---
## Signif. codes:  0 '***' 0.001 '**' 0.01 '*' 0.05 '.' 0.1 ' ' 1
```

```
ITTPModel<-lmer(log(InsulinTtP)~Dose*Treatment + (1|Subject),data=barD
ata)
anova(ITTPModel)
```

```
## Type III Analysis of Variance Table with Satterthwaite's method
##                 Sum Sq Mean Sq NumDF  DenDF F value Pr(>F)
## Dose           0.17750 0.17750     1 64.237  1.1012 0.2979
## Treatment      1.51685 0.75842     2 64.084  4.7053 0.0124 *
## Dose:Treatment 0.58789 0.29395     2 64.041  1.8237 0.1697
## ---
## Signif. codes:  0 '***' 0.001 '**' 0.01 '*' 0.05 '.' 0.1 ' ' 1
```

```
GBTPModel<-lmer(log(GlucoseBtP)~Dose*Treatment + (1|Subject),data=barD
ata)
anova(GBTPModel)
```

```
## Type III Analysis of Variance Table with Satterthwaite's method
##                 Sum Sq Mean Sq NumDF  DenDF F value    Pr(>F)
## Dose            3.3823  3.3823     1 63.653  8.5059  0.004884 **
## Treatment      10.3732  5.1866     2 63.456 13.0433 1.798e-05 ***
## Dose:Treatment  0.0478  0.0239     2 63.396  0.0601  0.941738
## ---
## Signif. codes:  0 '***' 0.001 '**' 0.01 '*' 0.05 '.' 0.1 ' ' 1
```

```
IBTPModel<-lmer(log(InsulinBtP)~Dose*Treatment + (1|Subject),data=barD
ata)
anova(IBTPModel)
```

```
## Type III Analysis of Variance Table with Satterthwaite's method
##                 Sum Sq Mean Sq NumDF  DenDF F value    Pr(>F)
## Dose           3.5232  3.5232     1 62.897 20.9967 2.235e-05 ***
## Treatment      4.7453  2.3726     2 62.807 14.1398 8.512e-06 ***
## Dose:Treatment 0.0534  0.0267     2 62.777  0.1592    0.8532
## ---
## Signif. codes:  0 '***' 0.001 '**' 0.01 '*' 0.05 '.' 0.1 ' ' 1
```

```
HOMAModel<-lmer(log(HOMA.IR)~Dose*Treatment + (1|Subject),data=barData
)
anova(HOMAModel)
```

```
## Type III Analysis of Variance Table with Satterthwaite's method
##                  Sum Sq Mean Sq NumDF  DenDF F value  Pr(>F)
## Dose           0.85220 0.85220     1 68.016  6.2873 0.01455 *
## Treatment      0.06163 0.03081     2 68.025  0.2273 0.79726
## Dose:Treatment 0.10759 0.05379     2 68.025  0.3969 0.67397
## ---
## Signif. codes:  0 '***' 0.001 '**' 0.01 '*' 0.05 '.' 0.1 ' ' 1
```

```
InsGluModel<-lmer(log(Ins.Glu)~Dose*Treatment + (1|Subject),data=barDa
ta)
anova(InsGluModel)
```

```
## Type III Analysis of Variance Table with Satterthwaite's method
##                  Sum Sq Mean Sq NumDF  DenDF F value Pr(>F)
## Dose           0.00791 0.00791     1 63.609  0.0094 0.9232
## Treatment      2.81320 1.40660     2 63.471  1.6660 0.1972
## Dose:Treatment 1.05504 0.52752     2 63.429  0.6248 0.5386
```
